# A serum TNFR2-based model effectively predicates preoperative microvascular invasion and stratifies the tumor recurrence risk in hepatocellular carcinoma

**DOI:** 10.1186/s12876-025-04152-y

**Published:** 2025-08-27

**Authors:** Peiru Zhang, Jianyong Zhuo, Huigang Li, Modan Yang, Jinyan Chen, Xudong Yang, Chenghao Cao, Shusen Zheng, Xiao Xu, Di Lu

**Affiliations:** 1 Zhejiang Chinese Medical University, Hangzhou, Zhejiang China; 2 Department of Hepatobiliary and Pancreatic Surgery, Affiliated Hangzhou First People’s Hospital, Westlake University, Hangzhou, Zhejiang China; 3 Zhejiang University School of Medicine, Hangzhou, Zhejiang China; 4 Hangzhou Normal University, Hangzhou, Zhejiang China; 5 Department of Hepatobiliary and Pancreatic Surgery, Shulan (Hangzhou) Hospital, Hangzhou, Zhejiang China; 6 Department of Hepatobiliary and Pancreatic Surgery, The First Affiliated Hospital, Zhejiang University School of Medicine, Hangzhou, Zhejiang China; 7 Hangzhou Medical College, Hangzhou, Zhejiang China; 8 NHC Key Laboratory of Combined Multi-organ Transplantation, Hangzhou, Zhejiang China

**Keywords:** Hepatocellular carcinoma, Microvascular invasion, Soluble tumor necrosis factor receptor-2, Milan criteria, Tumor recurrence

## Abstract

**Aim:**

Microvascular invasion (MVI) is a key risk factor for hepatocellular carcinoma (HCC) recurrence. There is a lack of methods to diagnose MVI preoperatively. The objective of this study was to develop a model for preoperative prediction of MVI in HCC.

**Method:**

The training cohort data were obtained from our previous study. One hundred and fourteen liver transplant patients with HCC were enrolled for validation. The serum level of soluble tumor necrosis factor receptor-2 (sTNFR2) was detected by ELISA. The Kaplan-Meier method was used for survival analysis. The multivariate logistic regression analysis was used to identify independent predictors of MVI, and a nomogram was constructed for visualization.

**Result:**

The recipients with MVI had significantly poorer outcomes than those without MVI both in the training cohort (*n* = 83, *P* < 0.001) and the validation cohort (*P* < 0.001). The inflammatory profiling from the training cohort data indicated that the serum level of B-cell activating factor (*P* = 0.014) and sTNFR2 (*P* = 0.013) significantly elevated, and the serum level of osteocalcin (*P* = 0.002) decreased in patients with MVI. Multivariate logistic analysis showed that the Milan criteria and the serum sTNFR2 were independent predictors for the presence of MVI, and a nomogram was constructed. The nomogram demonstrated an area under the receiver operating characteristic curve (AUROC) of 0.821 for MVI and distinct stratification for tumor recurrence (*P* < 0.001). Furthermore, the data in the validation cohort revealed an acceptable discriminative ability of confirmed MVI (AUROC = 0.702) and a notable discriminating capability for tumor recurrence (*P* = 0.043).

**Conclusion:**

The non-invasive model based on sTNFR2 could effectively predict preoperative MVI in HCC. And the nomogram could discriminate the tumor recurrence risk for HCC.

**Supplementary Information:**

The online version contains supplementary material available at 10.1186/s12876-025-04152-y.

## Background

Hepatocellular carcinoma (HCC) is the most common type of primary liver cancer, accounting for approximately 90% of all cases [1]. Approximately 400,000 new HCC cases are recorded in China every year, accounting for 55% of all cases worldwide. In Chian, HCC ranks fifth in cancer incidence and second in mortality rate [2]. Surgical resection and liver transplantation are potentially curative treatments for patients with HCC [3, 4], but postoperative recurrence imposes significant limitations on long-term patient survival. Approximately 70% of the patients experience recurrence within 5 years of surgical resection [5–8].

Microvascular invasion (MVI) has been identified as a high-risk factor for tumor recurrence [9, 10]. Multiple national guidelines for HCC indicate MVI as an important reference for estimating the risk of liver cancer recurrence and metastasis, selecting treatment options, and recommending the use of relevant models to predict MVI before surgery, which can provide a reference for subsequent treatment [11–13]. MVI-positive HCC is characterized by a high recurrence rate, increased likelihood of extrahepatic metastasis, and rapid disease progression [14–19]. MVI is a histopathological feature of micrometastases that can be observed in 15.0–57.1% of surgical specimens [10, 20]. However, methods for determining MVI preoperatively are currently lacking. Preoperative knowledge of MVI is critical in determining the most appropriate treatment regimen. Therefore, developing a preoperative prediction model is essential.

Serological markers and imaging indicators such as tumor size, number, boundary, and envelope are closely related to the preoperative prediction of microtubular carcinoma embolism. In recent years, many researchers have sought to determine the preoperative imaging features of patients with MVI to build predictive models [21–23]. However, the limitations of algorithms and devices have made them not universally applicable. In terms of serological markers, many researchers have realized that relevant indicators have a significant predictive value for HCC combined with MVI [24, 25]. Tumor necrosis factor (TNF), a multifunctional cytokine, promotes the growth, invasion, and metastasis of HCC [11, 26]. Therefore, we hypothesized that TNF associated with MVI. TNF exerts diverse biological functions by binding to two cognate membrane receptors, tumor necrosis factor receptor (TNFR) 1 and TNFR2. TNFR2 promotes regulatory T cell (Treg) expansion and tumor cell proliferation by activating multiple classical pathways and creating an immunosuppressive microenvironment [27, 28]. sTNFR2, a soluble form of TNFR2, synergistically promotes tumor growth and enhances tumor invasiveness with TNFR2. Serological indicators are indispensable for screening preoperative noninvasive predictors of MVI.

Therefore, we utilized various assays, including imaging, serum inflammatory profiles, and biomarkers to identify the most relevant factors. Consequently, we developed a multidimensional predictive model based on sTNFR2 and the Milan criteria. This model can be used to ascertain quickly and precisely whether a patient has MVI preoperatively, thereby providing clinicians with a valuable reference point for the development of treatment plans.

## Methods

### Samples

For the training cohort, the data was obtained from our previous study [29]. The data included demographic parameters, tumor burden, blood biochemical markers, cytokine profiling, and survival data.

For the validation cohort, the serums were collected from Shulan (Hangzhou) Hospital, from January 2015 to December 2020 and were enrolled in this retrospective study. All patients were fully informed and provided written informed consent before surgery.

All HCCs were confirmed by pathology examinations, and the MVI was evaluated by two independent pathologists, maintaining consistency of assessment criteria.

### The detection of serum sTNFR2

The serum expression of sTNFR2 in the validation cohort was tested by ELISA (BYabsciences, Cat#BY-EH110973). In brief, add the enzyme reagent to the standard and sample, followed by incubation. Then add the termination solution to read the OD value and calculate the result.

### The detection of TNFR2 in tumor

Immunohistochemistry (IHC) was performed using anti-TNFR2 antibodies (Abcam, ab109322, 1:100 dilution, Cambridge, UK). The IHC staining was scored by two independent pathologists who were blinded to the patients’ clinical characteristics. Refer to this study for assessment methods [26].

## Statistical analysis

The statistical analyses were performed using SPSS version 26.0 (IBMCorp, Armonk, NY, USA), GraphPad Prism version 9 (La Jolla, CA, USA), and R software version 4.0.2, with packages: “Hmisc”, “rms”, “zoo”. The chi-square test was utilized to compare the categorical variables. The Students’ t-test was utilized to compared the continuous variable conforming to a normal distribution. With regard to the matter of continuous variables that are not normally distributed, such as AFP, AST, etc., the recommended course of action is to take the logarithm with e as the base. The data was then subjected to a Shapiro-Wilk test, which confirmed that it conformed to a normal distribution. The non-normal continuous variables were tested nonparametric. Normally distributed variables were presented as a mean ± standard deviation, non-normally distributed variables were presented as the medians (interquartile range), and categorical variables were expressed as numbers (percentages). Multivariate logistic regression was used to estimate hazard ratios with 95% confidence intervals (CIs). The Kaplan-Meier method with the log-rank test was employed for survival analysis.

## Results

### Patients with MVI had poorer outcomes than patients without MVI

**Fig. 1:**
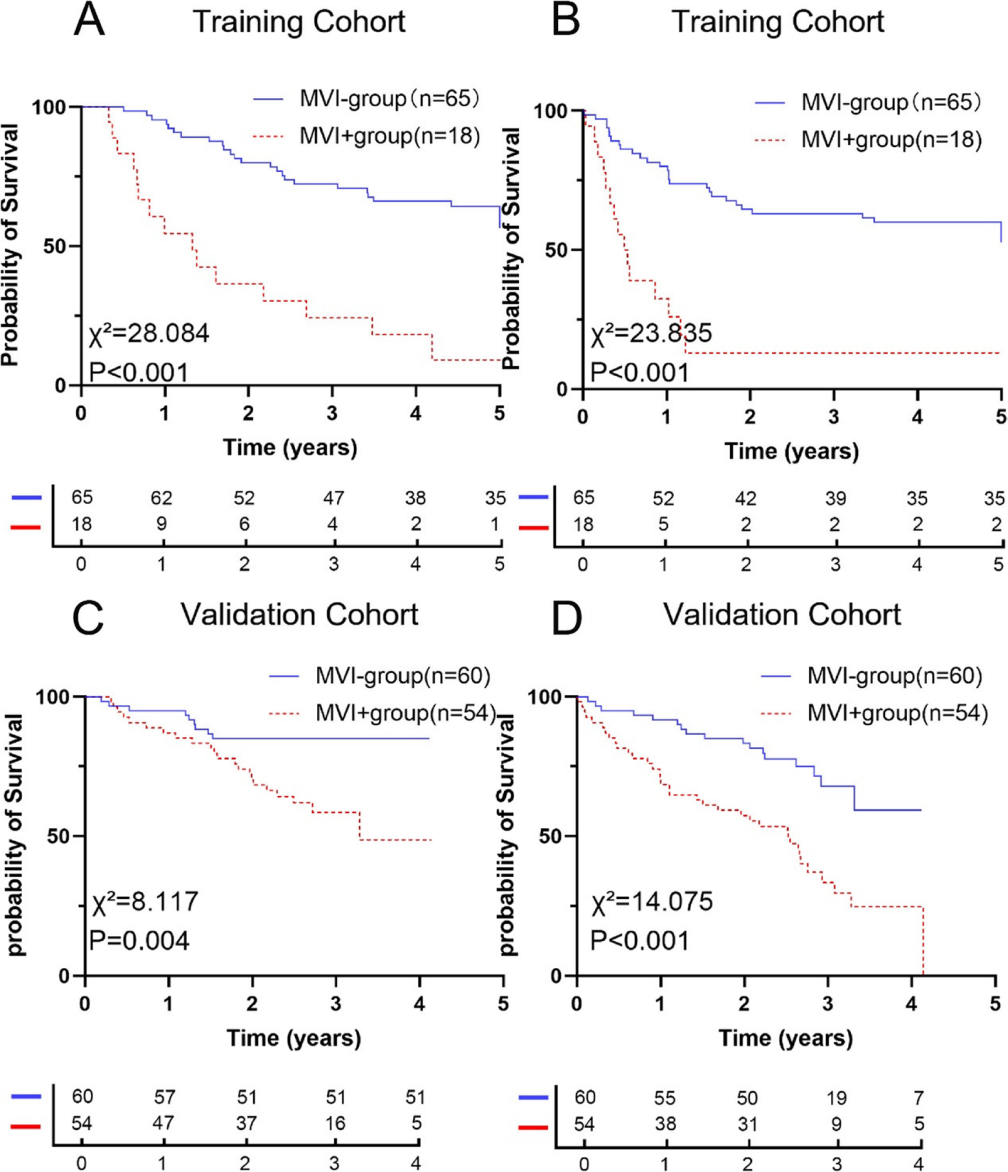
Relationship between MVI and post-transplant prognosis of HCC patients undergoing liver transplantation. (**A**,**B**) In the training cohort, Kaplan-Meier survival curves showing OS (*P*<0.001) (**A**) and RFS (*P*<0.001) (**B**) in MVI- group (*n*=65) and MVI+ group (*n*=18); (**C**,**D**) In validation cohort, Kaplan-Meier survival curves showing OS (P=0.004) (C) and RFS (*P*<0.001) (**D**) in MVI- group (*n*=60) and MVI+ group (*n*=54)

To screen the population for suitability for the study, a total of 83 patients from the aforementioned study cohort [29], were used as the training cohort (S-Figure 1). Patients with MVI exhibit a worse prognosis than those without MVI. Based on the presence or absence of MVI, we stratified the patients into MVI-positive (MVI+, *n* = 18) and MVI-negative (MVI−, *n* = 65) groups in the training cohort. The 1-, 3-, and 5-year overall survival (OS) rates in the MVI + and MVI − groups were 54.5%, 24.2%, and 9.1% vs. 95.4%, 72.3%, and 64.3%, respectively (*P* < 0.001, Fig. 1A). The 1-, 3-, and 5-year recurrence-free survival (RFS) rates for patients in the MVI + and MVI − groups were 32.4%, 13.0%, and 13.0% vs. 80.0%, 63.1%, and 60.0%, respectively (*P* < 0.001, Fig. 1B).

### MVI-related demographic parameters

To identify the certain detectors for the pre-operative presence of MVI, we compared the demographic parameters, tumor pathological characteristics, blood biochemical markers, and inflammatory factors between the MVI + and MVI − groups in the training cohort. As shown in Table 1, the MVI + and MVI − groups exhibited comparable baseline characteristics, with no statistically significant differences in mean age (52.56 ± 9.60 years vs. 52.06 ± 7.92years; *P* = 0.109) or prevalence of liver cirrhosis (100% vs. 96.92%; *P* = 0.451). Similarly, the HBsAg positivity rates did not differ significantly between the MVI + and MVI − groups (100% vs. 92.3%; *P* = 0.513).

**Table 1:** Baseline of MVI positive and negative patients in the training cohort

	MVI- group (*n* = 65)	MVI + group (*n* = 18)	*P* value
Gender (*n*, % female)	6 (9.23%)	2 (11.11%)	0.811
Age (years)	52.06 ± 7.92^1^	52.56 ± 9.60^1^	0.109 0.513
HBsAg positive (*n*, %)	60 (92.31%)	18 (100%)	
Live cirrhosis (*n*, %)	63 (96.92%)	18 (100%)	0.451
Maximum tumor diameter (*n*, % >5 cm)	18(27.7%)	11(61.1%)	0.019
Tumor number (*n*, % >3)	10(15.4%)	10(55.6%)	0.001
Differentiation			0.660
poorly differentiated	16	6	
Moderate/well differentiated	49	12	
Survival (years)	4.55 (2.78–6.32) ^2^	1.16 (0.05–2.28) ^2^	< 0.001
MELD	10.64 ± 7.78^1^	12.63 ± 10.47^1^	0.139
Milan criteria (*n*, % beyond)	27 (41.54%)	16 (88.89%)	0.001
Preoperative treatment (*n*, %)	43 (66.15%)	14 (77.77%)	0.513
AFP (ug/L) ^*^	3.96 ± 2.48^1^	5.65 ± 3.46^1^	0.066
AST (U/L) ^*^	3.74 ± 0.65^1^	4.29 ± 0.99^1^	0.037
ALT (U/L) ^*^	3.54 ± 0.77^1^	3.59 ± 0.76^1^	0.805
GGT (U/L) ^*^	4.36 ± 0.88^1^	4.69 ± 0.86^1^	0.162
Albumin (g/L)	36.82 ± 5.02^1^	37.11 ± 5.31^1^	0.827
Globulin (g/L)	29.81 ± 6.26^1^	29.34 ± 6.83^1^	0.784
Leukocyte (10^9^/L)	3.80 (2.85–5.75) ^2^	4.25 (3.18–6.63) ^2^	0.389
Lymphocyte (10^9^/L)	0.90 (0.60–1.30) ^2^	0.93 (0.50–1.17) ^2^	0.607
Neutrophil (10^9^/L)	2.20 (1.40–3.85) ^2^	2.90 (1.90-5.03^2^)	0.176
Monocyte (10^9^/L)	0.38 (0.24–0.58) ^2^	0.37 (0.31–0.71) ^2^	0.603
Platelet (10^9^/L)	78.00(52.50–122.00) ^2^	74.00 (45.00-140.25) ^2^	0.603

^1^Data are mean ± SD

^2^Median

^*^logarithmic

### MVI-related tumor burden

The pathological characteristics of tumors are closely related to MVI [30–32]. The MVI + group had a higher percentage of having more than three tumors and having the largest tumor diameter of > 5 cm than the MVI − group (55.6% vs. 15.4%, *P* = 0.001; 61.1% vs. 27.7%, *P* = 0.019). Moreover, the MVI + group had more tumors beyond the Milan criteria than the MVI − group (88.89% vs. 41.54%, *P* = 0.001). However, the degree of tumor differentiation did not significantly differ between the two groups (*P* = 0.660).

### MVI-related blood biochemical markers and inflammatory factor

Blood biochemical markers and inflammatory factors are commonly used as predictors of MVI in medical research. Recipients with MVI had higher aspartate aminotransferase (AST) than those without MVI (*P* = 0.037). But the levels of alpha-fetoprotein (AFP), alanine aminotransferase (ALT) and gamma-glutamyl transferase (GGT) did not differ between the two groups *P* = 0.066, *P* = 0.805, and *P* = 0.162, respectively). We also compared several blood-related indicators. No differences in leukocyte, lymphocyte, neutrophil, monocyte, or platelet counts are shown in Table 1.

Furthermore, analysis of inflammatory factor expression showed that the MVI + group had higher expression levels of B-cell activating factor (BAFF) and sTNFR2 (*P* = 0.014and *P* = 0.013, respectively) and lower osteocalcin (*P* = 0.002; Table 2).

**Table 2:** Inflammatory spectrum of MVI positive and negative patients in the training cohort

Inflammatory spectrum (pg/ml)	MVI- group (*n* = 65)	MVI + group (*n* = 18)	*P* value
APRIL ^*^	11.62 ± 0.45^1^	11.68 ± 0.56^1^	0.651
BAFF ^*^	9.26 ± 0.62^1^	9.70 ± 0.80^1^	0.014
sCD30 ^*^	6.13 ± 0.67^1^	6.40 ± 0.48^1^	0.125
sCD163 ^*^	12.01 ± 0.52^1^	12.21 ± 0.59^1^	0.176
Chitinase3like1 ^*^	9.25 ± 0.40^1^	9.32 ± 0.47^1^	0.583
gp130 ^*^	10.25 ± 0.46^1^	10.42 ± 0.51^1^	0.178
IFNα2 ^*^	4.09 ± 0.38^1^	4.08 ± 0.46^1^	0.967
IFNβ^*^	3.46 ± 1.19^1^	3.84 ± 0.71^1^	0.098
IFNγ^*^	4.02 ± 0.44^1^	4.09 ± 0.53^1^	0.547
IL2 ^*^	2.78 ± 0.67^1^	2.91 ± 0.52^1^	0.364
sIL6Rα^*^	8.02 ± 0.52^1^	8.08 ± 0.66^1^	0.710
IL8 ^*^	4.09 ± 0.41^1^	4.19 ± 0.42^1^	0.367
IL10 ^*^	1.40 ± 0.11^1^	1.43 ± 0.11^1^	0.409
IL11 ^*^	1.61 ± 0.64^1^	1.71 ± 0.48^1^	0.519
IL12p40 ^*^	4.48 ± 0.46^1^	4.57 ± 0.48^1^	0.473
IL12p70 ^*^	0.45 ± 1.15^1^	0.50 ± 0.78^1^	0.869
IL19 ^*^	4.23 ± 0.68^1^	4.23 ± 0.77^1^	0.971
IL20 ^*^	1.38 ± 0.38^1^	1.33 ± 0.39^1^	0.857
IL22 ^*^	3.39 ± 0.92^1^	3.48 ± 1.15^1^	0.725
IL26 ^*^	4.05 ± 0.38^1^	4.09 ± 0.44^1^	0.712
IL27p28 ^*^	2.87 ± 0.95^1^	2.84 ± 0.69^1^	0.903
IL28A ^*^	2.94 ± 0.48^1^	2.98 ± 0.71^1^	0.788
IL29 ^*^	5.40 ± 0.41^1^	5.48 ± 0.47^1^	0.444
IL32 ^*^	3.72 ± 0.74^1^	3.65 ± 1.19^1^	0.757
IL34 ^*^	5.93 ± 0.93^1^	6.00 ± 0.93^1^	0.758
IL35 ^*^	5.80 ± 0.47^1^	5.88 ± 0.52^1^	0.525
LIGHT ^*^	2.52 ± 1.03^1^	2.82 ± 0.97^1^	0.272
MMP1 ^*^	7.40 ± 0.59^1^	7.53 ± 0.61^1^	0.395
MMP2 ^*^	10.92 ± 0.57^1^	10.82 ± 0.63^1^	0.515
MMP3 ^*^	9.13 ± 0.54^1^	9.27 ± 0.58^1^	0.352
osteocalcin ^*^	7.97 ± 0.69^1^	7.36 ± 0.80^1^	0.002
osteopontin ^*^	11.00 ± 0.48^1^	11.23 ± 0.61^1^	0.091
Pentraxin3 ^*^	8.06 ± 0.76^1^	8.27 ± 0.73^1^	0.307
sTNFR1 ^*^	7.88 ± 0.73^1^	8.20 ± 0.58^1^	0.090
sTNFR2 ^*^	7.21 ± 0.56^1^	7.58 ± 0.53^1^	0.013
TSLP ^*^	4.82 ± 0.39^1^	4.91 ± 0.40^1^	0.392
TWEAK ^*^	5.71 ± 0.47^1^	5.59 ± 0.56^1^	0.336
IL6 ^*^	1.06 ± 0.88^1^	1.16 ± 0.87^1^	0.677

^1^Data are mean ± SD

^*^logarithmic

### Establishment of a nomogram according to the Milan criteria and sTNFR2

**Fig. 2:**
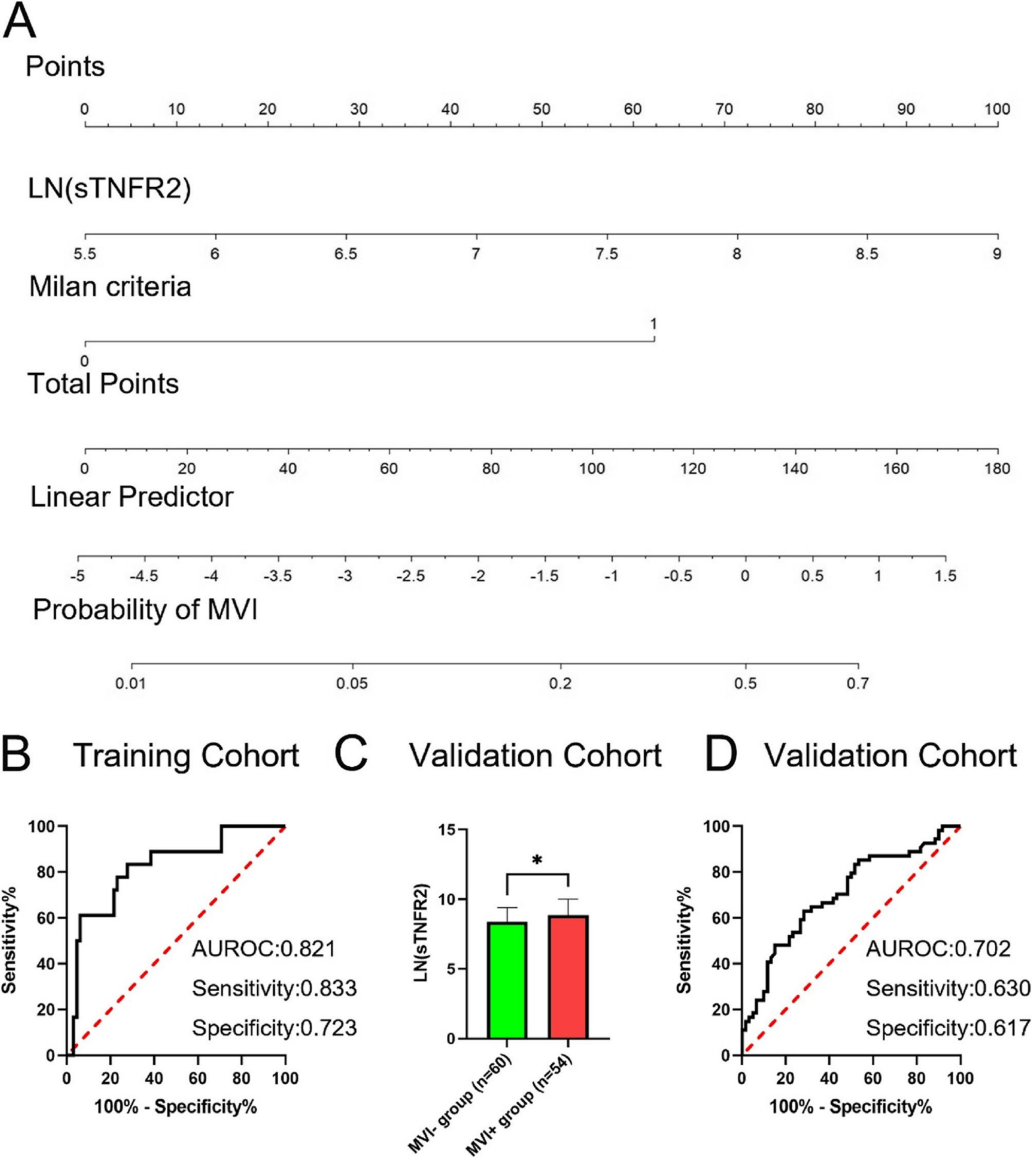
Nomogram for predicting MVI and validation of its efficacy. Nomogram model for predicting MVI of HCC patients (**A**); ROC curve (**B**, **D**) of the model in training and validation cohorts; Expression of sTNFR2 in validation cohort (**C**), *, *P*<0.05

Modelling was performed based on the results of a previous analysis. All variables with *P* < 0.1 included BAFF, interferon-β, sTNFR1, sTNFR2, osteocalcin, osteopontin, AFP, AST, and Milan criteria were entered into the analysis. Multivariate logistic regression analysis showed that the Milan criteria and sTNFR2 levels were independent predictors of MVI (Table 3). A nomogram was established based on the independent predictors (Fig. 2A). The predictive value of the area under the receiver operating characteristic curve (AUROC) was 0.821, sensitivity was 0.833 and specificity was 0.723, with a concordance index (C-index) of 0.821 (95%CI: 0.796, 0.845) (Fig. 2B).

**Table 3:** Multivariate logistic regression analysis of the variables

Variables	HR (95%CI)	*P* value
BAFF	1.768 (0.538–5.814)	0.348
IFNβ	0.736 (0.336–1.615)	0.445
osteocalcin	1.089 (0.340–3.484)	0.886
osteopontin	0.574 (0.118–2.805)	0.493
sTNFR1	0.767 (0.130–4.520)	0.769
sTNFR2	2.960 (1.042–8.404)	0.042
AFP	1.186 (0.962–1.461)	0.110
AST	1.599 (0.726–3.522)	0.244
Milan criteria	10.689 (2.215–51.579)	0.003

### Baseline differences between the training and validation cohorts

The training and validation cohorts had 18 and 54 MVI positives respectively (21.7% vs. 47.4%, *P* < 0.001). A total of 97.6% and 84.2% of the recipients in the training and validation cohorts, respectively, had liver cirrhosis (*P* = 0.010), and the HBsAg positivity rates were 93.9% and 80.7%, respectively (*P* = 0.014). Thus, the training cohort had a smaller percentage of patients with more than three tumors than the validation cohort (*P* = 0.019). The training cohort had lower model for end-stage liver disease (MELD) scores than the validation cohort (11 ± 0.9 vs. 32.4 ± 0.89, *P* < 0.001). However, tumor size did not significantly differ between the two groups (*P* = 0.901). Nevertheless, the proportion of patients who exceeded the Milan criteria was comparable between the two cohorts (51.81% vs. 54.39%, *P* = 0.831), although this was not significantly different between the two cohorts (Table 4).

**Table 4:** Patient baselines for training cohort and validation cohort

	Training Cohort (*n* = 83)	Validation Cohort (*n* = 114)	*P* value
Gender (*n*, % female)	8 (9.63%)	6 (5.26%)	0.368
Age (years)	52.17 ± 8.25^1^	53.57 ± 9.533^1^	0.243
HBsAg positive (*n*, %)	78 (93.98%)	92 (80.70%)	0.014
Live cirrhosis (*n*, %)	81 (97.59%)	96 (84.21%)	0.010
Maximum tumor diameter (*n*, % >5 cm)	29(34.9%)	42(36.8%)	0.901
Tumor number (*n*, % >3)	20(24.1%)	47(41.2%)	0.019
MVI positive (*n*, %)	18 (21.7%)	54 (47.4%)	< 0.001
Survival(years)	3.94(2.05–5.83)^2^	2.54 (2.08-3)^2^	< 0.001
MELD	11 ± 0.9^1^	32.4 ± 0.89^1^	< 0.001
Milan criteria (*n*, % beyond)	43 (51.81%)	62 (54.39%)	0.831
preoperative treatment (*n*, %)	57 (68.67%)	65 (57.02%)	0.130

‘*n*’ represents the number of times the event occurs, and ‘%’ represents its proportion, results using chi-square

^1^Data are mean ± SD, results using Students’ t-test

^2^Median, results using nonparametric

### Prognosis of the validation cohort and the application of nomogram

As with the training cohort, MVI − patients in the validation cohort had a worse prognosis. The 1- and 3-year OS rates were 87.0% and 58.4% in the MVI + group, and 95.0% and 85.0% in the MVI − group (*P* = 0.004, Fig. 1 C). The 1- and 3-year RFS rates for patients in the MVI + and MVI − groups were 70.4% and 33.3% vs. 91.7% and 67.9%, respectively (*P* < 0.001, Fig. 1D).

Furthermore, the sTNFR2 expression was examined in the validation cohort. In accordance with the findings of the training cohort, the sTNFR2 expression level in the MVI + group from the validation cohort was higher than that in the MVI − group (Fig. 2C). When this model was applied to the validation cohort, the predictive value of the AUROC, sensitivity, and specificity were 0.702, 0.630, and 0.617, respectively (Fig. 2D), indicating good prediction efficiency. Therefore, this nomogram was more effective in predicting MVI occurrence.

### Risk stratification of MVI based on the nomogram and recurrence analysis

**Fig. 3:**
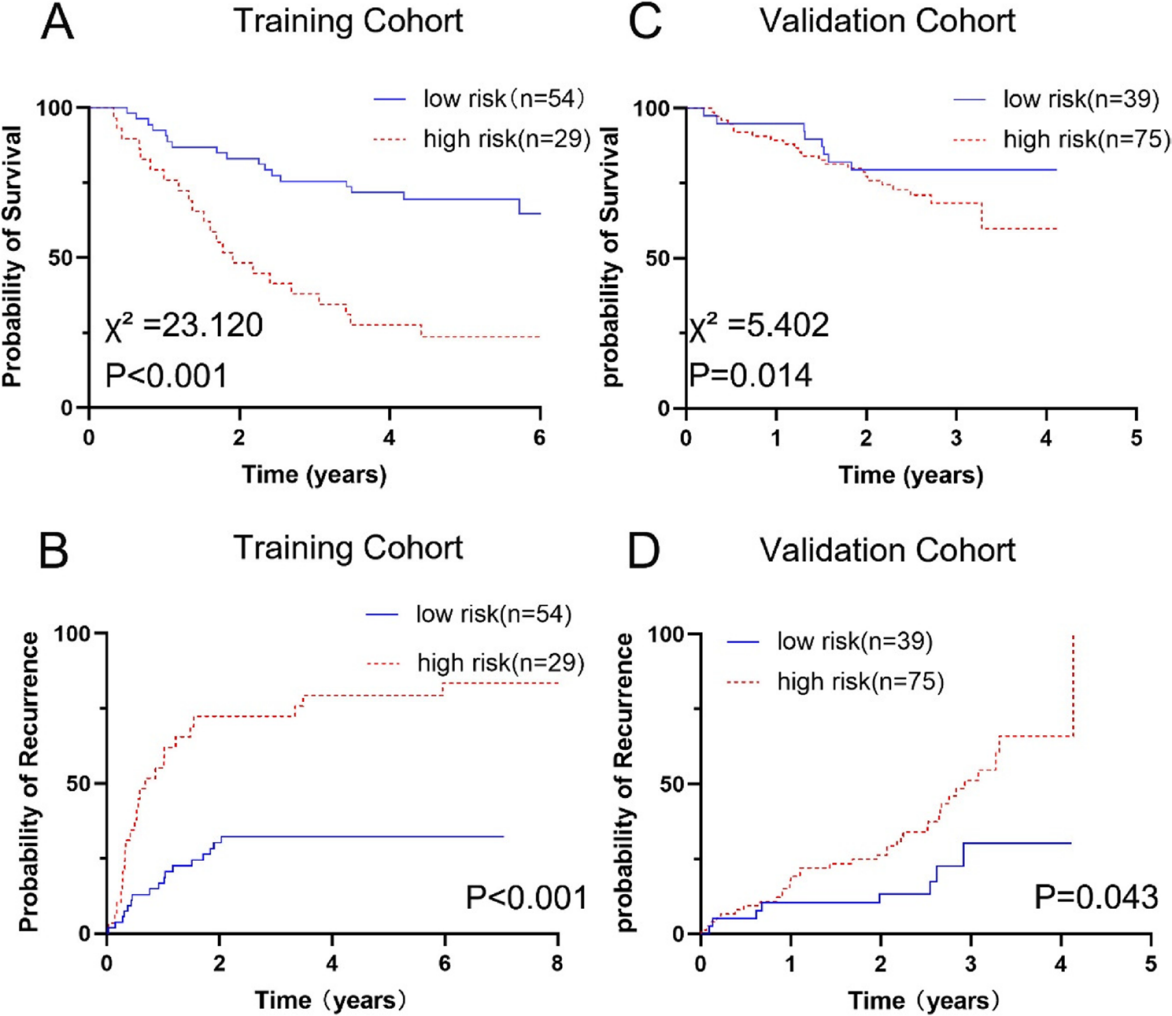
Prediction of prognosis and tumor recurrence according to the degree of MVI risk. Kaplan–Meier curves of RFS for patients with different risk scores in training cohort (**A**) and validation cohort (**C**); Cumulative postoperative recurrence rate curve for patients with different risk scores in training cohort (**B**) and validation cohort (**D**)

The optimal cut-off value was calculated according to the nomogram from training cohort, and the cohort was divided into the high and low-risk groups to predict prognosis and tumor recurrence. As shown in Fig. 3, the 1-, 3-, and 5-year RFS rates for patients in the high- and low-risk groups were 48.0%, 32.0%, and 24.0% vs. 79.2%, 61.6%, and 61.6%, respectively, in the training cohort (*P* < 0.001, Fig. 3A). The 1-, 3-, and 5-year recurrence rates in the high-risk group were 52.0%, 68.0%, and 76.0%, respectively, and 20.2%, 36.9%, and 36.9%, respectively, in the low-risk group (*P* < 0.001, Fig. 3B). The risk of recurrence was 2.89 times higher for those in the high-risk group than in the low-risk group (hazard ratio [HR] = 2.89, *P* = 0.001). And the optimal cut-off value was obtained using a validation cohort. In the validation cohort, the high-risk group demonstrated significantly lower 1- and 3-year RFS rates (78.4% vs. 87.5% and 44.2% vs. 63.3%, respectively; *P* = 0.014) and higher recurrence rates (18.0% vs. 10.1% at 1 year; 52.1% vs. 28.9% at 3 years; *P* = 0.043) than the low-risk group (Fig. 3C, D). And the risk of relapse was 1.99 times higher in the high-risk group than in the low-risk group (HR = 1.99, *P* = 0.036).

## Discussion

Patients with MVI had poor prognosis and a high recurrence rate. Therefore, predicting MVI preoperatively is crucial.

We compared the demographics, imaging, and biomarkers factors between MVI + and MVI − patients in the training cohort. Multivariate logistic regression analysis showed that the Milan criteria and sTNFR2 levels were independent risk factors for MVI. A predictive model was constructed based on these two features.

However, discrepancies were observed between the training and validation cohorts (Table 4). The training cohort exhibited a higher proportion of hepatitis B virus infection and cirrhosis cases than the validation cohort. Nevertheless, the number of tumors and MELD scores of the patients in the training cohort were lower than those in the validation cohort. Therefore, the literature was exhaustively reviewed to ascertain whether these differences had an impact on the results. The detection rate of MVI was positively correlated with the tumor size. The incidence rates of MVI in tumors with maximum diameters of < 3 cm, 3–5 cm, and > 5 cm were 17–40.6%, 31–42.5% and,46.2–93.4%, respectively [33–45]. Hepatitis B has been widely reported as an independent risk factor for MVI [30–32]. Meanwhile, the remaining variance factors not associated with MVI occurrence. Therefore, these discrepancies do not have a significant impact on the outcomes. However, the two study cohorts overrepresented patients with cirrhosis and hepatitis B, and whether the model can be applied to cohorts of patients with other HCC characteristics remains unknown. Hence, we will expand the sample size to verify the generalizability.

As previously indicated, a robust correlation was observed between tumor size and MVI presence. The Milan criteria are as follows: a single tumor with a diameter of < 5 cm or fewer than three multiple tumors with a maximum diameter of < 3 cm, no macrovascular invasion, and no lymph node or extrahepatic metastases [46]. The Milan criteria encompass tumor size; in our study, they were more suitable for predicting MVI occurrence. Consequently, the Milan criteria were selected as predictors.

sTNFR2 is soluble in TNFR2 and associated with cardiovascular diseases. TNF is associated with HCC recurrence [47–49]. sTNFR2 is formed by the shedding of membrane-type TNFR2, which can neutralize TNF-α and stabilize transmembrane-type TNF signaling, and promotes the expansion and activation of Treg. In solid tumors, such as lung cancer and ovarian cancer, an increase in Tregs inhibits the anti-tumor activity of effector T cells, creating an immunosuppressive microenvironment. This immune escape state may provide favorable conditions for MVI. Elevated sTNFR2 levels indicate sustained activation of the TNFR2 pathway, promoting tumor cell proliferation, migration and vascular infiltration capacity via NF-κB, MAPK and other signaling pathways. When sTNFR2 levels exceed a certain threshold, the risk of renal vascular injury doubles, which also highlights the fact that high levels of sTNFR2 can damage blood vessels and easily lead to MVI [50]. Experiments have demonstrated that TNFR2 and sTNFR2 levels are positively correlated in most solid tumors [27, 28]. Our previous study has indicated that TNFR2 is a negative prognostic factor for liver cancer, and is highly correlated with lung metastasis after liver cancer surgery [26]. A random selection of several hepatocellular carcinoma tissues was subjected to IHC staining, which revealed that TNFR2 expression was elevated in the MVI + group relative to the MVI − group (S-Figure 2). In addition, recent studies have indicated that TNFR2 may be a useful biomarker for predicting the response to treatment with ICIs. Combining anti-PD-1 and anti-TNFR2 antibodies as a novel immunotherapy for the treatment of HCC can produce better efficacy [27, 28]. Therefore, this molecule is highly correlated with the metastatic aggressiveness of cancer and can be used as a relevant marker.

Both of these features are associated with MVI development or tumor recurrence; therefore, the model based on them has a high confidence level. In addition, the patient’s risk of developing MVI can be quickly assessed because of the shorter examination time. The training and validation cohorts demonstrated some inevitable baseline differences. Compared to the validation cohort, the training cohort had more patients with hepatitis B and cirrhosis but a lower MELD score. A literature search revealed a few studies on cirrhosis and MVI, and the relationship between the two remains unclear. Numerous studies have indicated hepatitis B as an independent risk factor of MVI [30–32]. The MELD score is one of the best models for predicting survival in patients with end-stage liver disease, and its relationship with MVI is also unclear. Therefore, the training cohort possesses enhanced precision in predicting factors associated with MVI occurrence. Further demonstrating the general applicability of the model, its predictions were better for both the training and validation cohorts.

In addition to the Milan criteria and sTNFR2, several indicators in the univariate analysis were strongly associated with MVI. Although they ultimately failed to be included in the model, they still have potential applications. High serum AFP levels demonstrated an association with MVI, poor differentiation, and prediction of tumor recurrence after hepatectomy and transplantation. Owing to the low sensitivity of AFP, which is normal in up to 40% of patients with HCC, AFP generally needs to be combined with other factors to increase its sensitivity [51–54]. In this study, AFP could not be included in the model because the difference in AFP levels of the patients was not significant, owing to the effects of both cirrhosis and hepatitis B. Similarly, significant univariate associations with MVI were observed for other inflammatory markers such as BAFF and osteocalcin. No studies have directly linked BAFF or osteocalcin levels to MVI. BAFF overexpression is closely associated with chronic inflammation, and the inflammatory environment can disrupt the vascular endothelial barrier and promote tumor cell invasion into the vasculature [55]. BAFF and sTNFR2 may covary by sharing downstream pathways such as NF-κB or JNK signaling. In multifactorial analyses, model adjustment for covariance attenuated the independent contribution of BAFF, whereas sTNFR2 retained its significance, owing to its closer proximity to the end-effect link. Osteocalcin inhibits the release of pro-inflammatory factors such as TNF-α and IL-6, and the MVI progression is highly dependent on the inflammatory microenvironment [56, 57]. Therefore, osteocalcin may indirectly reduce vascular invasion by inhibiting inflammation. To date, no study has reported on the relationship between IFN-β or sTNFR1 and MVI. IFN-β primarily exerts its anti-tumor effects by inhibiting angiogenesis and enhancing anti-tumor immune responses. Based on these effects, it can be hypothesized that IFN-β may also inhibit the occurrence of MVI. It is hypothesized that sTNFR1 exerts an indirect inhibitory effect on MVI, primarily through the neutralization of TNF-α activity, the maintenance of Miz1 protein stability, and the protection of the vascular endothelial barrier. In this study, the expression levels of these two inflammatory factors were found to be higher in the MVI + group, contradicting the previously postulated mechanism. However, the p-values were 0.098 and 0.090 respectively, indicating that the differences between the two groups were not significant. Such a difference could be due to issues with the assay or the degradation of the samples themselves.

This model can quickly and noninvasively predict the likelihood of MVI occurrence in the preoperative period. For positive results, clinicians require more delicate manipulations, such as the no-touch technique, during transplantation to avoid tumor dissemination [58]. Clinicians should not consider negative result lightly and should combine them with other test results to make comprehensive judgments. The results of the model are not 100% correct, and false results may still occur. Therefore, clinicians should combine all test results to determine their reliability. Besides, increasing the sample size to improve the accuracy of the model in subsequent studies and to collect data on the effects of preoperative, intraoperative and postoperative treatments on false-negative or false-positive results. This will help us to identify appropriate treatments and avoid deterioration of the patient’s condition. In addition, model-based risk stratification enables clinicians to provide a reference for subsequent treatment of patients. The similarity in postoperative treatment protocols between the two hospitals had a negligible effect on the results of postoperative recurrence risk stratification. Therefore, appropriate postoperative treatment based on risk stratification may reduce recurrence rates. However, the 3-year recurrence rate in the high-risk group was 50%, indicating that a 50% chance of not recurrence remains. However, the positive predictive value for recurrence is limited. Therefore, we plant to expand the study population and conduct prospective studies to further improve this model.

Most of the current predictive models are based on images. The drawbacks are that the tools are not easy to deploy on a large scale, the algorithms require hardware support, and some require dedicated developers whose practicality remains uncertain [21–23, 59]. Cucchetti et al. utilized four common clinical variables, including AFP, tumor number, size, and volume, to develop an artificial neural network model that accurately identified 91% of the MVI cases in the testing group [60]. However, utilizing this model is constrained by its reliance on specialized computer software, which may limit its practicality in routine clinical settings. Li et al. constructed a nomogram by incorporating contrast-enhanced magnetic resonance imaging data with hematological data, which achieved a relatively good predictive accuracy for MVI [32]. Nevertheless, as the model was based solely on a single-center study, its optimal cutoff value is not universally applicable.

The present study had some limitations. First, the sample size was small, and this should be increased in future studies. Serum tests for inflammatory factors in patients from the two hospitals were not perform simultaneously. Lastly, the kits used were inconsistent, which may have led to some differences in the results. Should the sample size be increased at a future date in order to validate this study, it will be necessary to determine harmonized test kits and testing times. The purpose of this will be to ensure that the results are not affected by the test kits and testing times.

## Conclusion

The model was utilized to identify factors associated with the occurrence of MVI through multidimensional fetching. The final findings indicated a correlation between the Milan criteria and sTNFR2.The non-invasive model based on Milan criteria and sTNFR2 level could effectively predict microvascular invasion in HCC before LT and could discriminate the risk of posttransplant tumor recurrence.

## Supplementary Information

Supplementary Material 1.

## Data Availability

The data generated in this study are not publicly available due to information that could compromise patient privacy or consent but are available upon reasonable request from the corresponding author.

## Abbreviations

AFP: alpha-fetoprotein
ALT: alanine aminotransferase
AST: aspartate aminotransferase
AUROC: area under the receiver operating characteristic curve
BAFF: B-cell activating factor
C-Index: concordance Index
MELD: Model for end-stage liver disease
GGT: gamma-glutamyl transferase
HBV: hepatitis B virus
HCC: hepatocellular carcinoma
HR: Hazard Ratio
IFNβ: interferon-β
IHC: immunohistochemistry
LT: liver transplantation
MVI: Microvascular invasion
OS: Overall survival
RFS: Recurrence-free survival
sTNFR1: soluble tumor necrosis factor receptor-1
sTNFR2: soluble tumor necrosis factor receptor-2
Treg: regulatory T cell
