# Supplementary material for: A serum TNFR2-based model effectively predicates preoperative microvascular invasion and stratifies the tumor recurrence risk in hepatocellular carcinoma
Source: BMC Gastroenterol. 2025 Aug 27;25:622. doi: 10.1186/s12876-025-04152-y (PMC12392594; doi:10.1186/s12876-025-04152-y)
Supplement: Supplementary file 1 — Supplementary Material 1. [file 12876_2025_4152_MOESM1_ESM.docx]

**Figure 1. PRISMA flowchart**


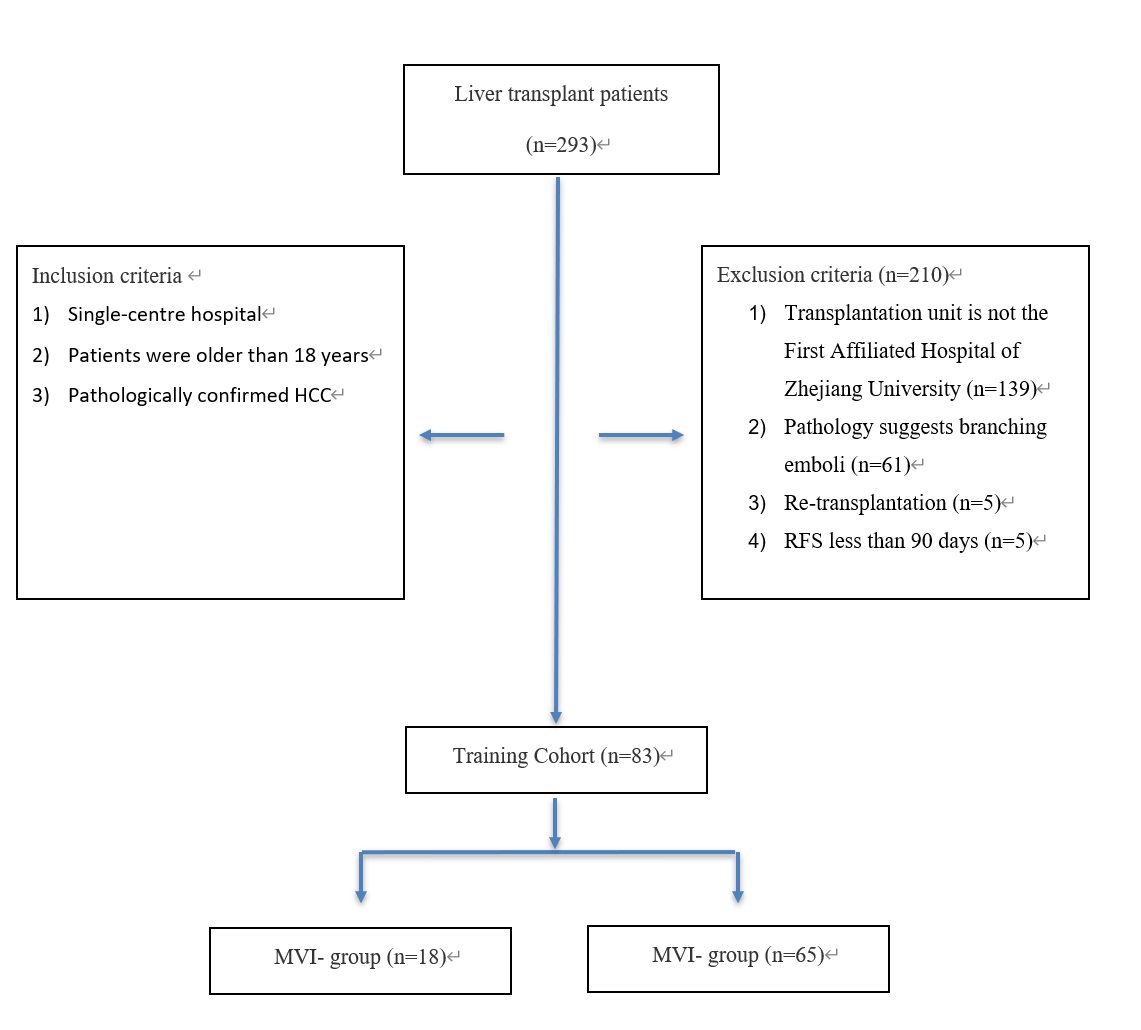


**Figure 2. TNFR2 expression in patients with MVI-negative and MVI-positive**


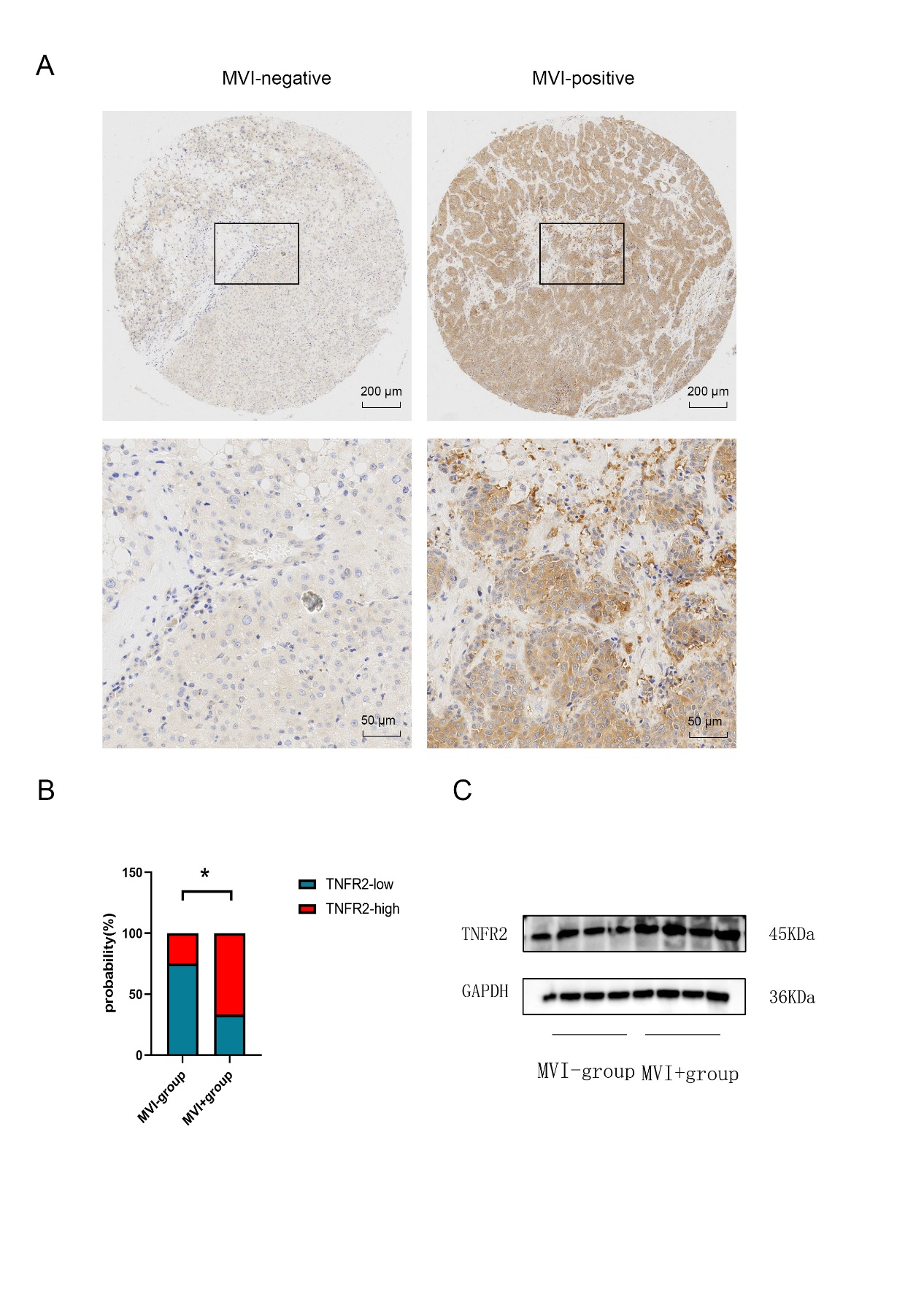


(A) Representative maps of TNFR2 in hepatocellular carcinoma tissues from MVI+ and MVI- hepatocellular carcinoma patients; (B) Percentage of MVI+ and MVI- patients with positive TNFR2 expression, *, P < 0.05; (C) TNFR2 expression in HCC samples from MVI- and MVI+ patients.
